# ATG9A loss confers resistance to trastuzumab via c-Cbl mediated Her2 degradation

**DOI:** 10.18632/oncotarget.8504

**Published:** 2016-03-30

**Authors:** Joao Nunes, Hua Zhang, Nicos Angelopoulos, Jyoti Chhetri, Clodia Osipo, Arnhild Grothey, Justin Stebbing, Georgios Giamas

**Affiliations:** ^1^ Department of Surgery and Cancer, Division of Cancer, Imperial College London, Hammersmith Hospital Campus, London, UK; ^2^ Department of Microbiology and Immunology, Cardinal Bernardin Cancer Center of Loyola University Chicago, Health Sciences Division, Maywood, Illinois, USA; ^3^ School of Life Sciences, University of Sussex, Brighton, UK

**Keywords:** breast cancer, trastuzumab, resistance, ATG9A, SILAC

## Abstract

Acquired or *de novo* resistance to trastuzumab remains a barrier to patient survival and mechanisms underlying this still remain unclear. Using stable isotope labelling by amino acids in cell culture (SILAC)-based quantitative proteomics to compare proteome profiles between trastuzumab sensitive/resistant cells, we identified autophagy related protein 9A (ATG9A) as a down-regulated protein in trastuzumab resistant cells (BT474-TR). Interestingly, ATG9A ectopic expression markedly decreased the proliferative ability of BT474-TR cells but not that of the parental line (BT474). This was accompanied by a reduction of Her2 protein levels and AKT phosphorylation (S473), as well as a decrease in Her2 stability, which was also observed in JIMT1 and MDA-453, naturally trastuzumab-resistant cells. In addition, ATG9A indirectly promoted c-Cbl recruitment to Her2 on T1112, a known c-Cbl docking site, leading to increased K63 Her2 polyubiquitination. Whereas silencing c-Cbl abrogated ATG9A repressive effects on Her2 and downstream PI3K/AKT signaling, its depletion restored BT474-TR proliferative rate. Taken together, our findings show for this first time that ATG9A loss in trastuzumab resistant cells allowed Her2 to escape from lysosomal targeted degradation through K63 poly-ubiquitination via c-Cbl. This study identifies ATG9A as a potentially druggable target to overcome resistance to anti-Her2 blockade.

## INTRODUCTION

Approximately 20–25% of breast cancers (BCa) have amplification/overexpression of human epidermal growth factor receptor 2 (ErbB2/Her2), a member of receptor tyrosine kinase family [1]. Homo or dimerisation with other family members such as Her3 results in phosphorylation of tyrosine residues within the cytoplasmic domain of the receptors and initiates signal transduction via the oncogenic PI3K/AKT and RAS/MAPK pathways [2, 3]. Therapeutic targeting of ErbB family members for cancer therapy includes tyrosine kinase inhibitors (TKIs) or recombinant monoclonal antibodies such as Trastuzumab (Tst). Binding of trastuzumab to the extracellular domain of HER2 inhibits the downstream signaling and improves survival in patients within both the metastatic and adjuvant settings in BCa [4–6]. However, despite initial clinical benefits, patients frequently relapse after therapy or display primary resistance to trastuzumab-based therapy indicating the presence *de novo* or acquired resistance [7, 8]. A number of mechanisms have been described to date, including hyperactivation of PI3K/AKT pathway [9], heterodimerization with other family members or compassion through an alternate receptor pathway [10], co-expression of the truncated p95Her2 receptor or loss Her2 expression [11]. Targeting these mechanisms have however proven to be insufficient to block progression of disease indicating a critical demand to prevent treatment failure.

Previously, autophagy has been indicated to play an important role in trastuzumab sensitivity in Her2 amplified breast cancer [12–14]. For instance, autophagy has been proposed to protect breast cancer cells from growth-inhibitory effects of trastuzumab [15] and autophagy blockage restored trastuzumab sensitivity in trastuzumab resistant cells [13]. However, the contribution of specific members of autophagy related protein family in the development of trastuzumab resistance and whether their functions are through autophagy signaling remain poorly understood. Autophagy related protein 9A (ATG9A) is the only known multi-pass transmembrane autophagy protein among over 30 ATG proteins identified to date. It has six conserved transmembrane domains and cytosolic N- and C-termini that are non-homologous between mammals and yeast. The ATG9A trafficking pathway remains unclear; to date ULK1, ATG13 and p38-interacting protein (p38IP) have been shown to interact with ATG9A. Under basal conditions, ATG9A is found in the trans-Golgi network, recycling and late endosomes whereas upon autophagy induction it reallocates to the periphery of the cell and co-localises with phagophore markers and autophagosomes. However, the function of ATG9A and its associated signaling in trastuzumab sensitivity in breast cancer were unknown.

In this study, we performed a quantitative proteomic analysis followed by mass spectrometry in established trastuzumab sensitive and resistant Her2 amplified breast cancer cells. Our results revealed that ATG9A protein levels are markedly reduced in trastuzumab resistant cells and restoring ATG9A levels can decrease Her2 stability and its protein levels. Strikingly, in trastuzumab resistant cells, ATG9A acts independently of autophagy; overexpression of ATG9A resultedd in targeted endosomal/lysosomal degradation of Her2 and consequently a decrease in resistance to trastuzumab. Our results are indicative of a unique role of ATG9A in trastuzumab resistant cells and suggest a potential significance of ATG9A as a target in patients when Her2 targeting drugs are no longer effective.

## RESULTS

### SILAC analysis reveals ATG9A as a potential regulator of trastuzumab resistance

**Figure 1:**
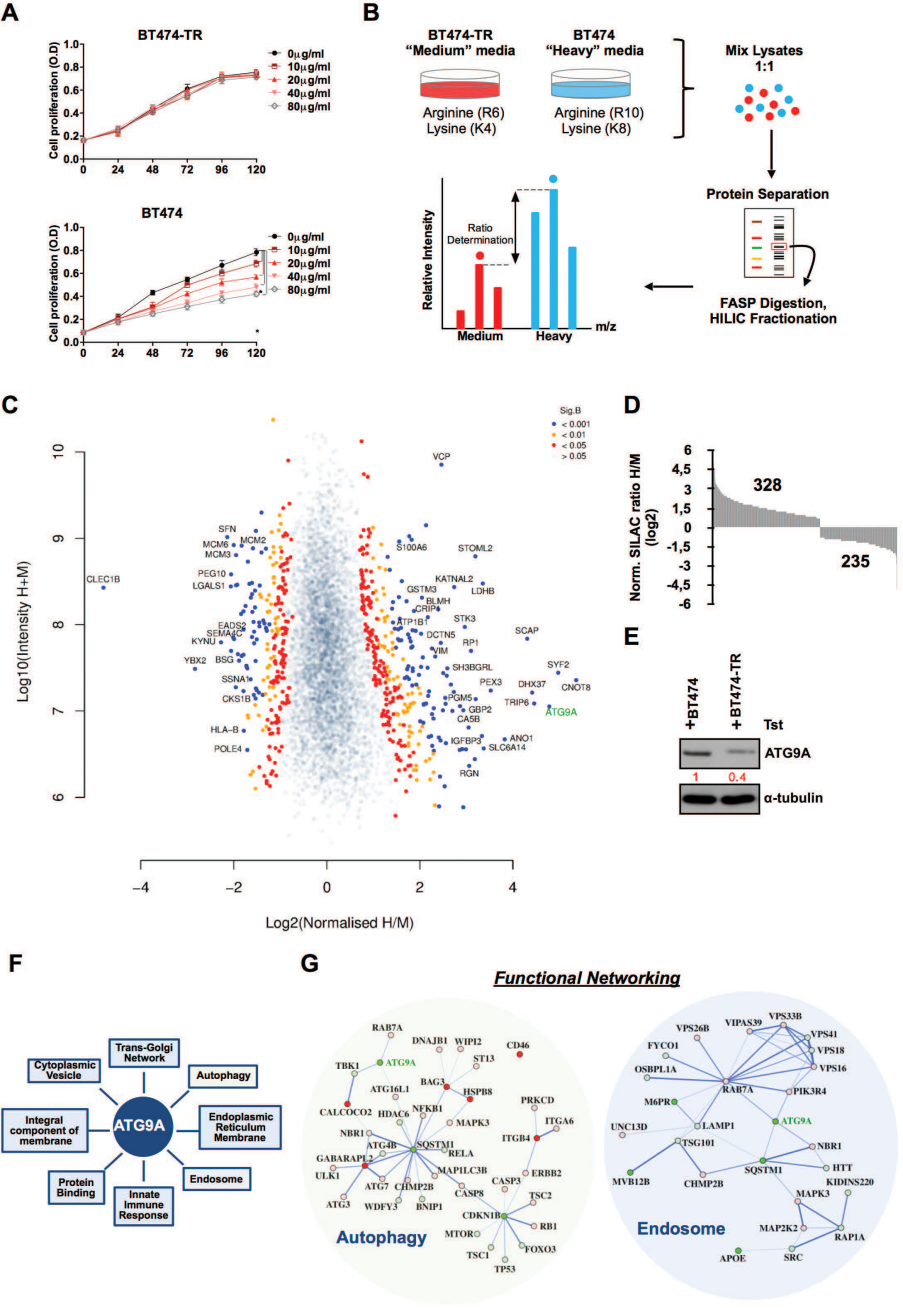
SILAC analysis reveals ATG9A as a potential regulator of trastuzumab resistance (**A**) Cell proliferation of BT474-TR and BT474 cells up to 5 days in the presence of increasing amounts of trastuzumab. (**B**) Experimental schematic outline of SILAC experiment. (**C**) Volcano scatter plot comparison of ratios for total proteins showing altered abundance in BT474 relative to BT474-TR in the presence of trastuzumab (20 μg/ml). Significance threshold was calculated by a B Significance Test (Sig B). (**D**) SILAC Log2 distribution of the significant B protein levels up or down regulated after SILAC analysis (**E**) Western blotting for ATG9A in BT474 and BT474-TR in the presence of trastuzumab (20 μg/ml). (**F**) Gene ontology terms in which ATG9A is classified. (**G**) SILAC data for the functional protein–protein interaction networks for Autophagy and Endosome GO terms. Protein nodes with red colour are up-regulated in BT474-TR, whereas green represents down-regulation in BT474-TR. Colour gradient indicates log2 ratio difference between BT474 and BT474-TR with lighter colour representing lower Log2 ratio and darker colour representing high Log2 ratio for each protein. Connective network lines thickness corresponds to the level of evidence of interaction. *P < 0.05 unpaired Student's *t* test.

To identify the differentially modulated proteome involved in trastuzumab resistance, we performed a quantitative proteomic analysis using metabolic labelling by SILAC and followed by LC-MS/MS. Firstly, BT474 parental and BT474-derived trastuzumab resistant cells (BT474-TR) were cultured in the presence of increasing amounts of trastuzumab to assess their proliferative response to the drug. Comparing to BT474 parental cells, BT474-TR cells did not respond to trastuzumab confirming the acquired resistance to the anti-Her2 monoclonal antibody (Figure 1A). Subsequently, parental BT474 and BT474 trastuzumab resistant (BT474-TR) cells were then grown for 7 cell divisions in R6K4 ‘medium’ or R10K8 ‘heavy’ medium, respectively. Lysates obtained from three independent experiments for each condition were mixed in order to decrease experimental error and increase biological significance (Figure 1B). A total of 5622 unique proteins were identified (Figure 1C, Supplementary Table S1), among which we identified 328 significantly up-regulated proteins in BT474 cells and 235 down-regulated in comparison with BT474-TR cells (Figure 1D). One of the most differentially dysregulated was ATG9A showing a pronounced down-regulation in parental BT474-TR cells (Log_2_ = 4.7864, sig.B 4.18E-18) and further validated by western blot (Figure 1E). GO analysis highlighted a variety of biological functions that ATG9a is mainly involved in, including autophagy and endosome regulation which our present study is focused on (Figure 1F). We then integrated our proteomic data with the STRING database. Of note, in agreement with previous studies, our analysis showed that whereas ATG9A was down-regulated, numerous central regulators of autophagy flux such MAP1LC3B (LC-3B), ULK1, ATG7, ATG3 and NF-KB, were up-regulated in BT474-TR. In addition, SQSTM1 a negative marker for autophagy induction was reduced in BT474-TR (Figure 1G). Further analysis also confirmed RAB7A and LAMP1 presence in endosome/late endosome compartment and suggested a possible interaction between ATG9A and RAB7A, which might be important for endo-lysosomal trafficking of receptor tyrosine kinases (Figure 1G).

To establish the relevance of ATG9A expression levels with clinical outcome in breast cancer, we then utilized the online survival analysis tool KM Plotter, which integrates massive gene expression data and survival information derived from more than 3,000 patients from databases including The Cancer Genome Atlas (TCGA) [16]. The correlations of ATG9A expression levels with relapse free survival (RFS) in all breast cancer patients as well as in the subgroup of Her2 amplified breast cancer were assessed. Our analysis showed that high ATG9A levels were associated with longer RFS in all breast cancer patients and interestingly, the same trend was also observed in patients with Her2 amplification (Figure S1). These findings indicate an important role of ATG9A in breast cancer and highlight a potential link between ATG9A and Her2.

### ATG9A overexpression reduces cell proliferation and increases apoptosis in trastuzumab resistance cells

**Figure 2:**
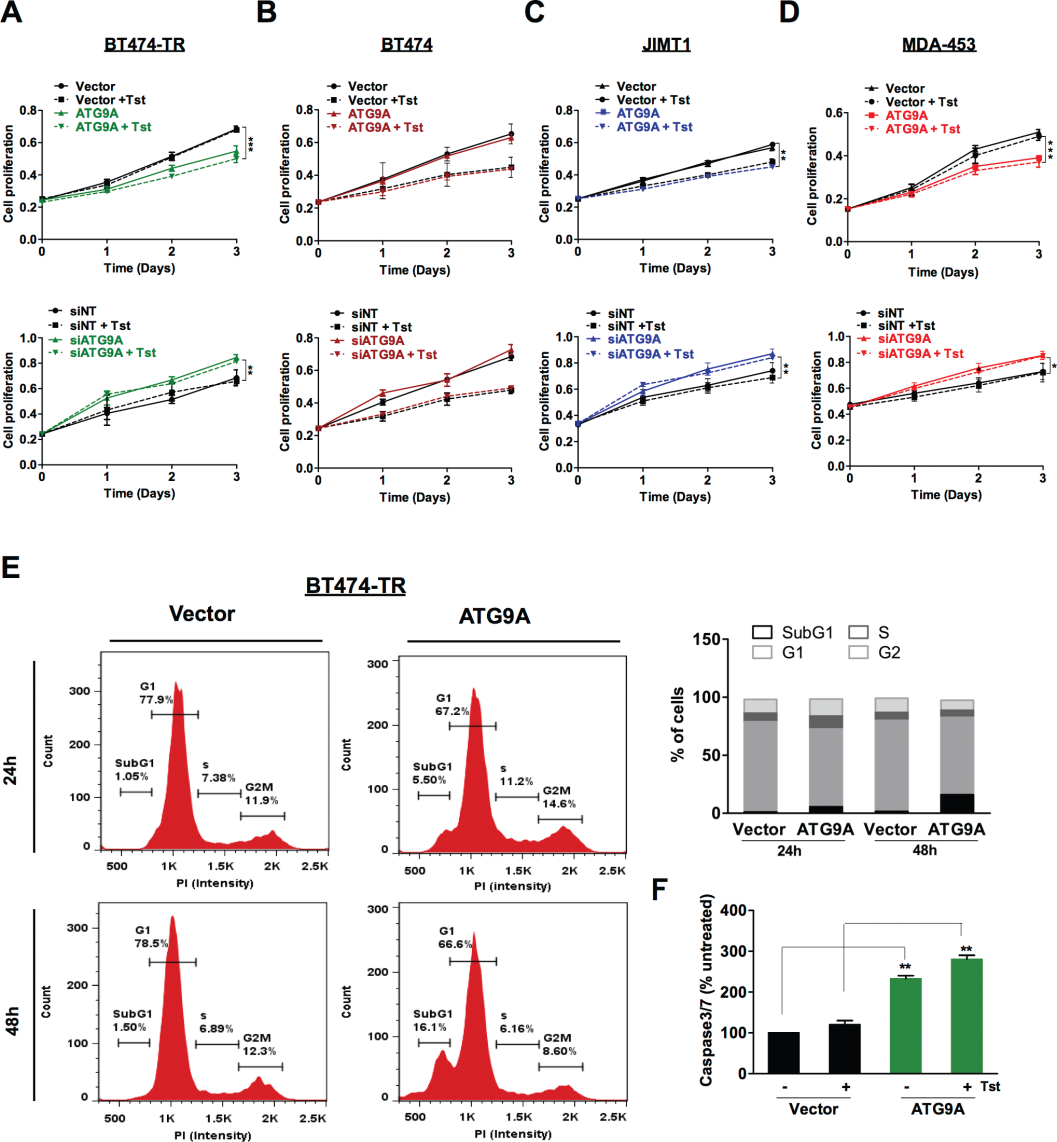
ATG9A overexpression represses cell proliferation and induces apoptosis in trastuzumab resistant cells (**A**–**D**) Effect of ATG9A overexpression (top graphs) and siATG9A (bottom graphs) on cell proliferation in (A) BT474-TR, (B) BT474, (C) JIMT1 and (D) MDA-453 cells in the presence or absence of trastuzumab (20 μg/ml). (**E**) Cell cycle analysis of BT474-TR transfected with ATG9A or empty vector in the presence of trastuzumab for 24 h and 48 hours. (**F**) caspase3/7 levels of BT474-TR transfected with ATG9A or empty vector in the presence or absence of trastuzumab for 48 hours. The data presented are means ± SEM from three independent experiments. *P < 0.05, **P < 0.01 and ***P < 0.001 unpaired Student's *t* test.

Oncogenic signaling driven by Her2 is strongly associated with increased progression and poor prognosis. Previous studies have implicated Her2 expression and down-stream signaling mediators as key regulators in cell proliferation and apoptosis [17, 18], prompt us to address whether ATG9A modulation could affect cellular proliferation. ATG9A overexpression in BT474-TR led to a significant decrease in cell proliferation as shown in Figure 2A (top graph), which was even more accentuated when trastuzumab was added to cells. Conversely, down-regulation of ATG9A by specific siRNA resulted in a significant increase in cell proliferation in BT474-TR cells, whereas trastuzumab addition did not change cell proliferative response (Figure 2A; bottom graph). Moreover, parental BT474 did not show proliferative response to ATG9A overexpression (Figure 2B; (top graph) or inhibition by siRNA (Figure 2B; bottom graph) suggesting that ATG9A repressive effect is trastuzumab-resistant specific. In support, JIMT1 and MDA-453 cell lines, which are innately resistant to trastuzumab, showed a markedly decrease in proliferation upon ATG9A overexpression (Figure 2C–2D; top graph), which the opposite effects (increased proliferation) were observed after ATG9A knockdown (Figure 2C–2D; bottom graph). Next, we sought to investigate whether ATG9A inhibitory-proliferative effect could be due to cell cycle dysregulation. Our data showed that ATG9A overexpression resulted in an increase in subG1 population without affecting other cell cycle phases, indicating an increase in apoptosis (Figure 2E). In support of this, caspase 3/7 levels were elevated upon ATG9A overexpression confirming the activation of apoptotic signaling (Figure 2F).

### ATG9A decreases Her2 protein levels and supresses PI3K/AKT signaling

**Figure 3:**
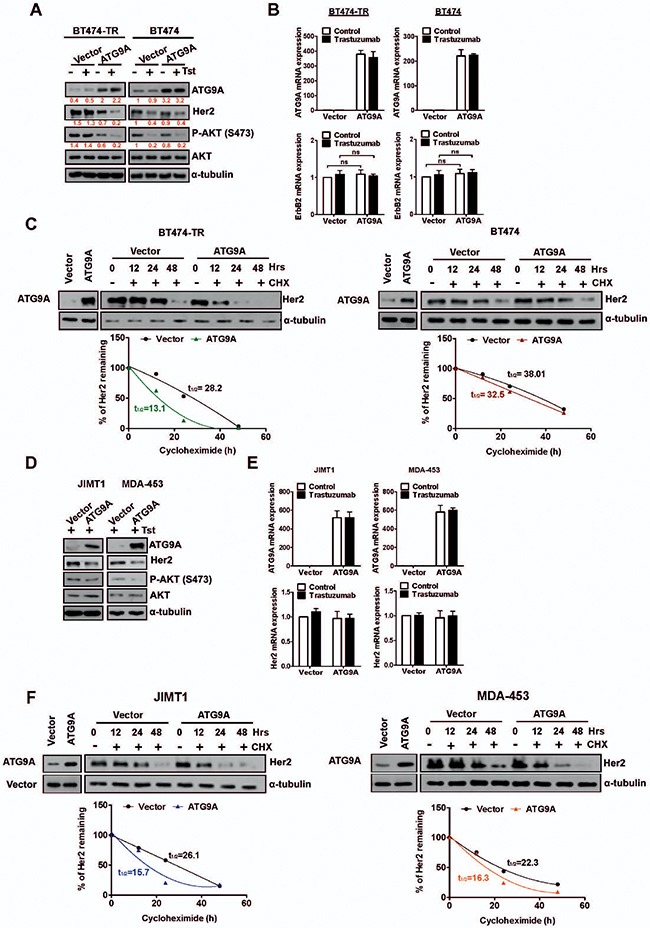
Her2 stability and protein levels are affected by ATG9A levels (**A**) Protein abundance of ATG9A, Her2, AKT and P27/KIP upon transfection with ATG9A or empty vector in the presence or absence of trastuzumab (20 μg/ml) in BT474-TR and BT474 (**B**) mRNA expression levels for ATG9A and Her2 upon transfection with ATG9A or empty vector in the presence or absence of trastuzumab (20 μg/ml) in BT474-TR and BT474. (**C**) BT474-TR and BT474 cells were treated with Cyclohexamide (100 μM) for up to 48 hours after overexpression of ATG9A or empty vector in the presence or absence of trastuzumab (20 μg/ml). Half-life of Her2 protein (t_1/2_) was calculated and compared between ATG9A and empty vector groups. (**D**) Protein abundance for ATG9A, Her2, AKT and (**E**) mRNA expression levels for ATG9A and Her2 in JIMT-1 and MDA-453 after transfection with ATG9A or empty vector in the presence or absence of trastuzumab (20 μg/ml). (**F**) JIMT1 and MDA-453 cells were treated with Cyclohexamide (100 μM) for up to 48 hours after overexpression of ATG9A or empty vector in the presence or absence of trastuzumab (20 μg/ml). Half-life of Her2 protein (t_1/2_) was calculated and compared between ATG9A and empty vector groups.

One of the well-established mechanism that impairs trastuzumab efficiency is the hyperactivation of the PI3K/ATK pathway [9], and AKT phosphorylation, a read-out for PI3K/AKT signaling, has been correlated with cell growth and survival [19]. We thus aimed to investigate whether the repressive proliferative effect on BT474-TR by ATG9A overexpression was through Her2 and its down-stream signaling mediators such as PI3K/AKT. Our data showed that Her2 protein basal levels were markedly decreased by ATG9A ectopic expression, an effect that was more accentuated with the addition of trastuzumab. Interestingly, this was observed in trastuzumab acquired resistant cells only (Figure 3A). In addition, AKT phosphorylation was also reduced in these cells indicating ATG9A's ability to negatively regulate PI3K/AKT signaling possible via Her2 inhibition. Moreover, when ATG9A was down-regulated by specific RNA interference, Her2 total protein levels were markedly increased in BT474-TR cells but not in parental BT474 cells. An increase in AKT phosphorylation was also proven to be specific for BT474-TR (Figure S2), as well as JIMT1 and MDA-453 cells (Figure S3). These findings indicate a specific role of ATG9A in regulating Her2 and downstream AKT signaling.

To further evaluate whether the effect of ATG9A on Her2 basal protein level was a reflection of a transcriptional repression, we performed a quantitative real-time polymerase chain reaction (RT-qPCR). ATG9A overexpression did not affect the Her2 transcript levels in either parental BT474 or acquired trastuzumab resistant cells (BT474-TR) (Figure 3B). Interestingly, Her2 regulated genes such as *S100A4*, *HSF1*, *COX2* and *IGFBP3* were found to be modulated by ATG9A overexpression at different levels (Supplementary Figures S4, S5). Again, this effect was only observed in BT474-TR, JIMT1 and MDA-453 but not in BT474 cells, in accordance with what was previously observed on Her2 protein levels. Overall, these data indicate that ATG9A might directly regulate Her2 at protein level, subsequently resulting in a decrease of Her2 stability and its down-stream effectors.

We then sought to elucidate whether ATG9A affects Her2 protein stability by blocking nascent protein translation with cyclohexamine (CHX). In BT474-TR, ATG9A overexpression triggered a decrease in Her2 protein levels (Figure 3C) with a half-life reduction from t_1/2_ = 28.2 to t_1/2_ = 13.1. In BT474 cells, a marginal decrease was observed (Vector t_1/2_ = 38.01, ATG9A t_1/2_ = 32.5) in Her2 basal protein levels upon ATG9A overexpression (Figure 3C) indicating that its effect on Her2 stabilization is more significant in trastuzumab resistant cells. These results support the hypothesis of a repressive mechanism on ATG9A to maintain high levels of the tyrosine kinase receptor as an oncogenic driver and highlight that ATG9A decrease in BT474-TR might contribute to trastuzumab resistance.

To confirm our findings in intrinsic trastuzumab resistant cells, we investigated whether ATG9A modulation would also affect Her2 protein and mRNA levels in JIMT1 and MDA-453 cells. Our data showed a significant decrease in Her2 protein and AKT phosphorylation in both JIMT1 and MDA-453 (Figure 3D). However, no change in Her2 mRNA levels was observed in both cell lines (Figure 3E). Upon CHX treatment, Her2 protein levels were more stable in control group comparing to ATG9A overexpression with a decrease of 40% and 27% in Her2 half-life protein levels for JIMT1 and MDA-453, respectively (Figure 3F).

### ATG9A regulates Her2 degradation via c-Cbl ubiquitin ligase

**Figure 4:**
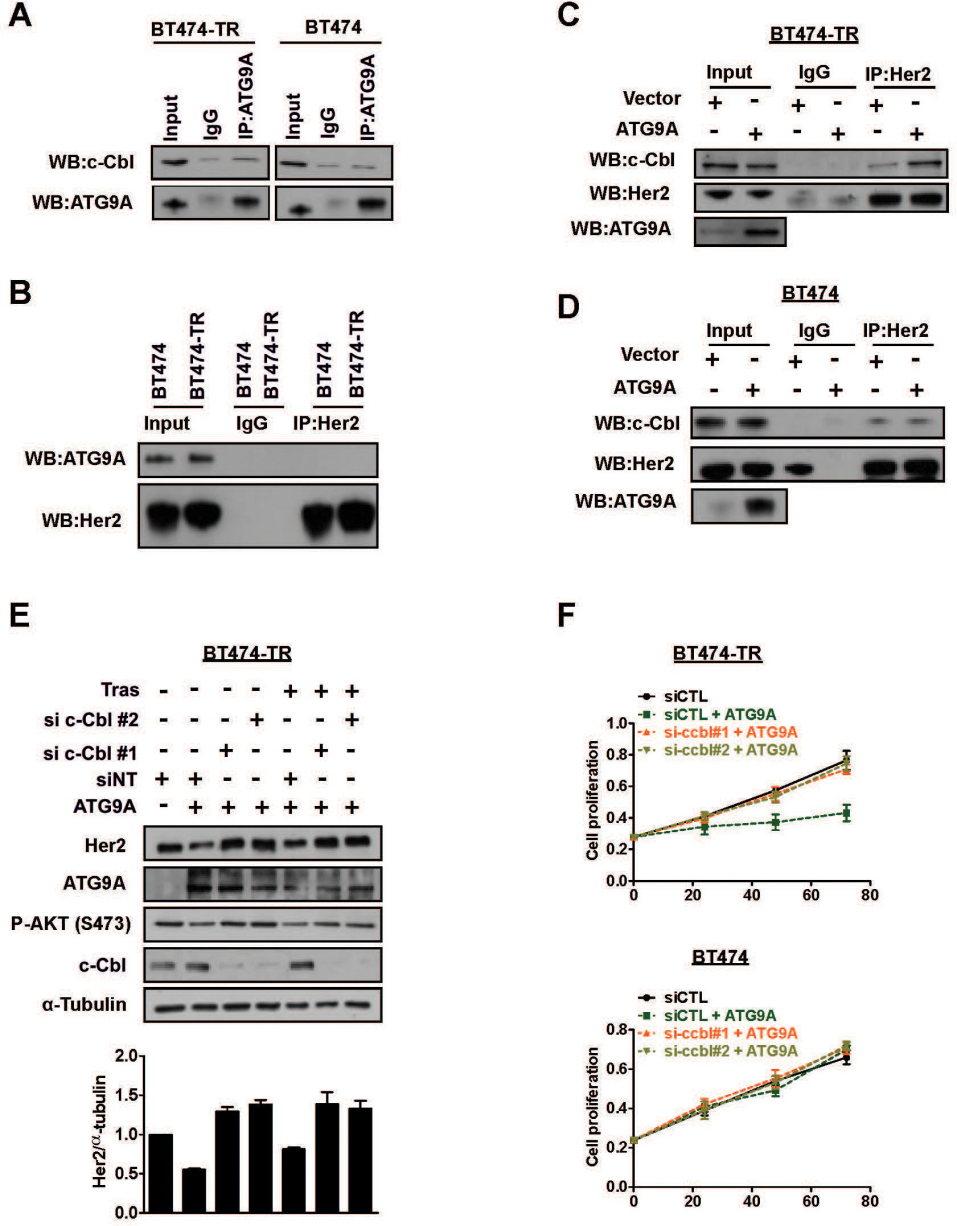
ATG9A induces Her2 protein degradation via c-Cbl E3 ubiquitin ligase (**A**) Immunoprecipitation of ATG9A and (**B**) Her2 followed by western blotting of indicated antibodies in BT474-TR and BT474 cells. IgG antibodies were used as controls. (**C**) Immunoprecipitation of Her2 in BT474-TR and (**D**) BT474 cells transfected with vector and ATG9A-encoding plasmid in the presence of trastuzumab (20 μg/ml) for 48 hours, followed by western blotting of indicated antibodies. (**E**) Western blotting for Her2, ATG9A, pAKT and c-Cbl upon sic-Cbl with or without ATG9A overexpression in the presence or absence of trastuzumab (20 μg/ml) in BT474-TR. Protein levels were analysed and quantified using Image J software. Her2 bands were normalised to α-tubulin. (**F**) BT474 and BT474-TR cell proliferation was analysed after siRNA silencing against c-Cbl (or a non-targeting siRNA (siCTL)), followed by overexpression of ATG9A (or control vector).

A previous study indicated c-Cbl, an E3 ubiquitin ligase, as an important regulator of Her2 degradation [20]. Thus, we sought to investigate whether ATG9A modulates Her2 protein levels and degradation via c-Cbl. Immunoprecipitation assays showed that there was no direct interaction between ATG9A and c-Cbl in both BT474-TR and BT474 (Figure 4A). In addition, ATG9A and Her2 are not binding partners in either cell line (Figure 4B). However, we observed an increase in c-Cbl capability to interact with Her2 upon ATG9A ectopic expression (Figure 4C), a phenomenon that was restricted to BT474-TR cells (Figure 4C–4D). Notably, targeting c-Cbl using siRNA significantly impaired ATG9A-induced degradation effect on Her2 protein levels and restored its abundance to basal conditions (Figure 4E). Likewise, PI3K/AKT signaling was also affected by c-Cbl targeted inhibition, an effect that appears to be trastuzumab independent, as no significant difference was observed in the presence or absence of the drug (Figure 4E). In addition, ATG9A-induced inhibition of cell proliferation in BT474-TR was suppressed by c-Cbl down-regulation (Figure 4F), an effect that was not observed in BT474 cells (Figure 4F). Overall, these results suggest that ATG9A indirectly promotes the formation of c-Cbl/Her2 complex and enhances Her2 degradation, consequently, affecting its downstream mediators such as PI3K/AKT. These effects of ATG9A seem to be mediated in a c-Cbl dependent manner.

### c-Cbl induces K63 Her2 polyUb redirecting for endosome/lysosome targeted degradation

ATG9A affects Her2 degradation via c-Cbl and previous work demonstrated that the binding of c-Cbl to tyrosine 1112 (Y1112) of Her2 leads to ubiquitination [21]. Moreover, Her2 polyubiquitin linkages via lysine 63 (K63) was associated with endosome trafficking to the lysosome leading to its degradation [22]. The ability of c-Cbl to induce K63 poly-ubiquitination (K63 polyUb) [23, 24] prompted us to investigate a potential role for ATG9A in affecting c-Cbl poly-ubiquitination of Her2 and lysosome targeted degradation.

**Figure 5:**
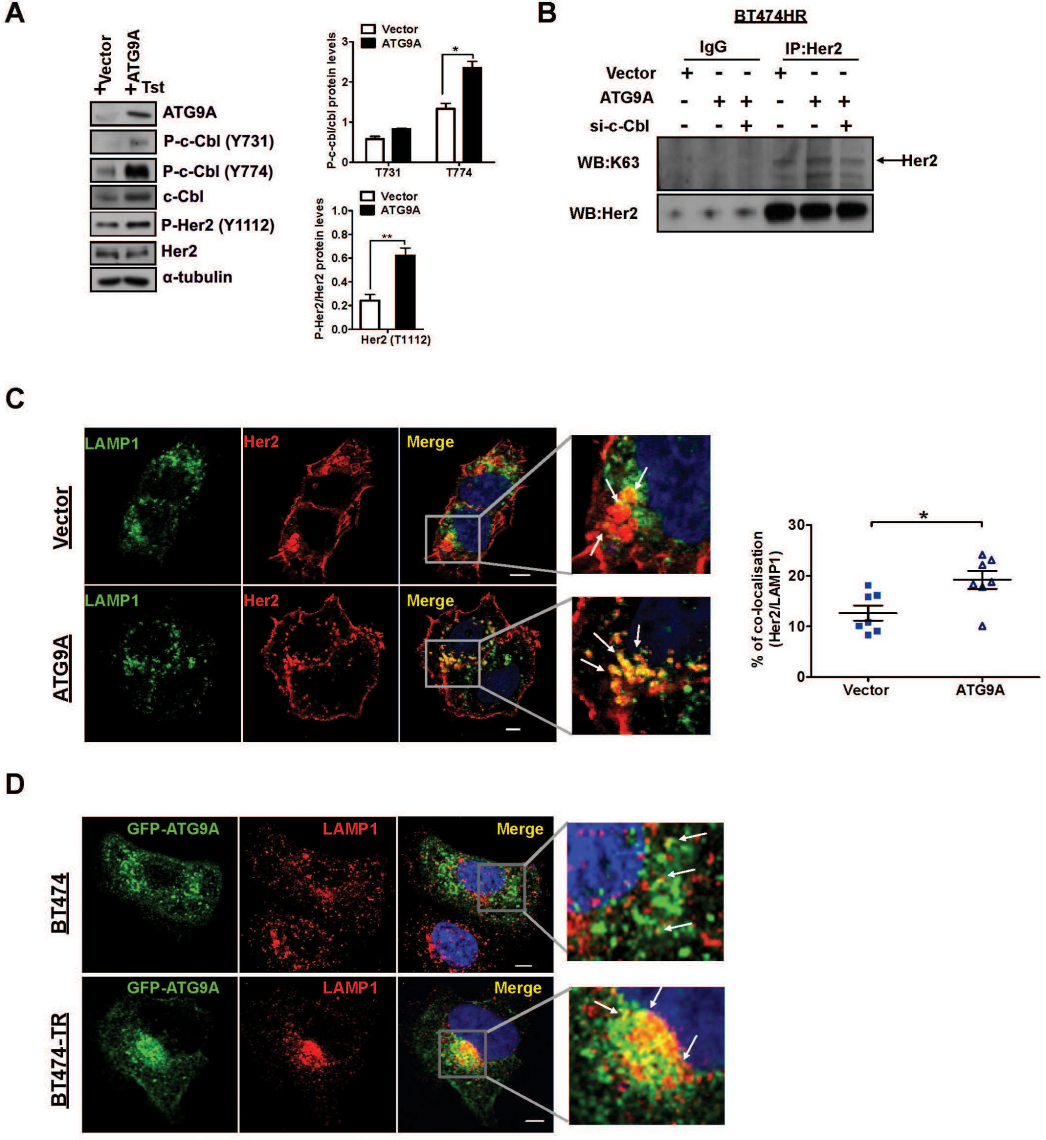
c-Cbl induces K63 Her2 polyUb redirecting for lysosomal targeted degradation (**A**) Effect of ATG9A on phosphorylation of c-Cbl in BT474-TR. Cells were transfected with vector and ATG9A-encoding plasmid in the presence of trastuzumab (20 μg/ml). Protein levels for ATG9A, c-Cbl total and phosphorylation sites Tyr 731 and 774, Her2 total and phosphorylated Tyr 1112 were quantified by Image J software. Bands were normalised to α-tubulin. (**B**) Immunoprecipitation of Her2 followed by western blotting for K63 and Her2 in BT474-TR cells transfected with siRNA against c-Cbl or siRNA, upon overexpressing ATG9A or control vector. IgG was used as control. (**C**) Immunofluorescence staining for LAMP1 and Her2 in BT474-TR cells upon transfection with vector or ATG9A-encoding plasmid in the presence of trastuzumab (20 μg/ml). Percentage of Her2/LAMP1 co-localisation was quantified using LEICA LAS AF lite software. (**D**) Immunofluorescence staining for GFP-ATG9A and LAMP1 in BT474 and BT474-TR transfected with ATG9A-GFP encoding plasmid in the presence of trastuzmab (20 μg/ml). Data is representative of three experiments. *P < 0.05, unpaired Student's *t* test.

Our data showed that ATG9A ectopic expression in BT474-TR induced a significant increase of c-Cbl phosphorylation at tyrosine 774 (Y774) and a moderate increase at tyrosine 731 (Y731), two known sites involved in the regulation of c-Cbl protein degradation (Figure 5A). More importantly, Her2 phosphorylation on the tyrosine 1112 (Y1112) was significantly increased, confirming ATG9A's ability to induce Her2 degradation via cbl activation (Figure 5A). We then sought to evaluate c-Cbl's capability to induce K63 polyUb of Her2. Her2 Immunoprecipitation in BT474-TR indicated an increase on K63 Her2 polyUb followed by ATG9A overexpression. This was abrogated by c-Cbl target inhibition by siRNA (Figure 5B), indicating a role for ATG9A in promoting Her2 degradation via c-Cbl induced K63 polyUb. Furthermore, we used two potent lysosome inhibitors (choroquine and bafilomycin) to examine whether ATG9A mediates Her2 lysosomal degradation. Both inhibitors were able to rescue ATG9A-induced Her2 degradation (Figure S6A).

In addition, MG132, a potent and selective inhibitor of the proteasome, failed to recover Her2 protein levels upon ATG9A overexpression (Figure S6B), reinforcing our hypothesis that ATG9A-induced Her2 degradation pathway is lysosome-dependent in trastuzumab resistant cells (BT474-TR). Despite the documented role of ATG9A in the phagosphore formation, there was no increase in autophagy influx as p62 and LC-3I/II protein levels were not affected by ectopic ATG9A expression (Figure S6C). The presence of Her2 within the lysosome was further confirmed by immunofluorescence, where LAMP1 (a marker for lysosomes) showed a significant increase of co-localisation with Her2 in BT474-TR upon ectopic expression of ATG9A (Figure 5C). Moreover, to further confirm this finding, we used another known regulator of EFGR trafficking and marker for late endosomes, RAB7. Immunofluorescence staining showed elevated co-localisation between Her2 and RAB7 upon ATG9A overexpression (Figure S7). An increased co-localisation between LAMP1 with ATG9A was also observed in BT474-TR cells in comparison with BT474 cells (Figure 5D). Collectively, our results suggest a role for ATG9A in Her2 endosomal/lysosome target degradation via c-Cbl K63 polyUb, which might be a unique signature in trastuzumab resistant breast cancer cells. This in turn maintains the Her2 oncogenic signaling and tumorigenic potential.

## DISCUSSION

Primary and acquired resistance to trastuzumab remains a major obstacle in the treatment of patients with Her2-amplified breast tumours [25]. It is therefore crucial to identify underlying mechanisms that could provide a more successful design of strategies to overcome this obstacle. Following this line of thought, we have used a pair of trastuzumab resistant cell line that was established by gradual exposure to trastuzumab (BT474-TR) and its parental BT474 as models in this study (Figure 1A). This model is close to the clinical setting where acquired resistance is mainly observed after prolonged administration of trastuzumab. Using SILAC-based quantitative proteomics, we compared the global proteomic profile of BT474-TR and parental BT474 cells to identify potential targets that are implicated in trastuzumab resistance development in BCa. We found that ATG9A was one of the most significantly down-regulated proteins in BT474-TR cells compared to sensitive parental BT474 (Figure 1C, 1E). As previous evidence suggests that ATG9A is involved in formation of the integral membrane during phagophore formation [40], it was reasonable to propose an autophagy promoting role for ATG9A. However, our data showed that ATG9A levels were down-regulated in trastuzumab resistance cells, whereas protein expression levels of autophagy markers such as SQSTM1/p62 indicated elevated autophagy flux (Figure 1G), which is in accordance with previous studies where autophagy influx was shown to be increased in trastuzumab resistance cells [13, 15]. It is possible that ATG9A might act as a negative regulator of autophagy in trastuzumab resistant cells only, but thus far we have no evidence supporting this.

On the other hand, our results uncovered an interesting role of ATG9A in contributing to trastuzumab resistance in BCa. First of all, overexpression of ATG9A was sufficient to decrease proliferation in both acquired resistant and naturally resistant cells (Figure 2A–2D), and the addition of trastuzumab had no significant repressive effect but did reduce Her2 levels to a further extend in acquired resistant cells (Figure 2A). Secondly, Her2 overexpression and hyperactivation of PI3K/AKT pathway has been correlated with trastuzumab resistance [18, 27, 28]. Here we showed that ATG9A was able to affect Her2-AKT driven signaling leading to a reduction in cell proliferation and an increase in apoptosis (Figure 1F).

More importantly, we revealed for the first time that ATG9A might act through c-Cbl to induce Her2 degradation in trastuzumab resistant cells but not sensitive cells. Our data demonstrated that ATG9A overexpression increased c-Cbl-Her2 interaction resulting in Her2 receptor degradation, an effect that appears to be independent of ATG9A and c-Cbl association (Figure 4B). We were also not able to detect any direct interaction between ATG9A and Her2 suggesting the existence of an unidentified mediator responsible for ATG9A-induced c-Cbl activation.

Previous studies showed that Y1112 is a docking site for Her2 ubiquitination by c-Cbl, an effect mediated by trastuzumab leading to receptor internalisation and consequent degradation [20]. In addition, c-Cbl is able to produce either K48 or K63 ubiquitin chains for Her2 polyubiquitin editing, resulting in proteasome or lysome dependent degradation [22]. In line with these findings, we observed that ATG9A overexpression markedly increased T1112 phosphorylation (Figure 5A) and K63 polyUb (Figure 5B) of Her2. Moreover, ATG9A was able to decrease Her2 half-life (Figure 3C) and to increase co-localisation between Her2 with RAB-7 and LAMP-1, two markers for endosome/lysosome trafficking, highlighting a new role for ATG9A in c-Cbl-induced degradation of Her2 via lysosomal degradation.

**Figure 6:**
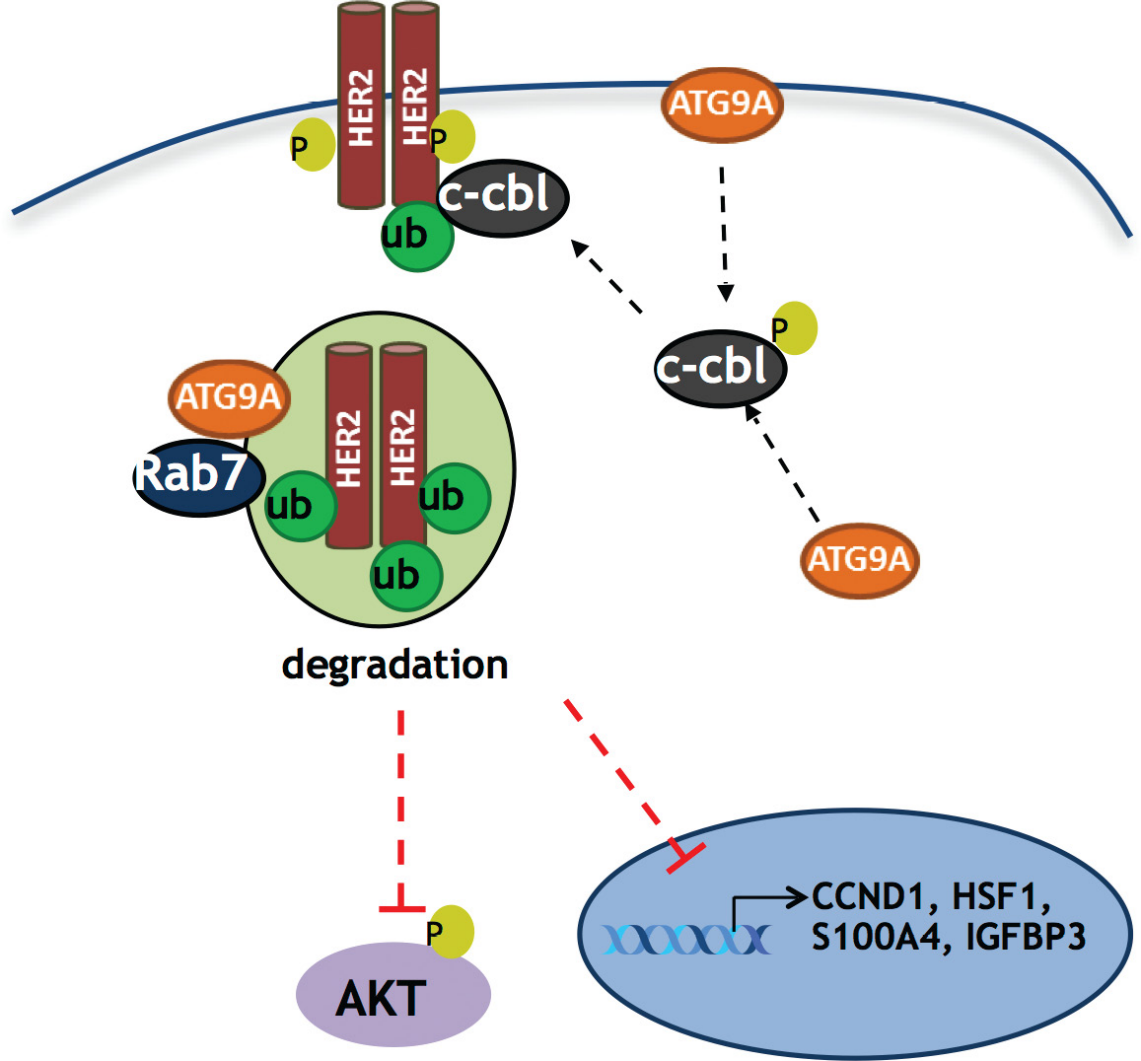
Schematic model of ATG9A's role in trastuzumab resistant cells ATG9A promotes c-Cbl recruitment to the receptor kinase Her2 on T1112, a known c-Cbl docking site, leading to an increase in Her2 poly-ubiquitination. This ultimately results in a decrease in Her2 protein levels, Her2 transcriptional activity and Her2 downstream PI3K/AKT signaling.

Taken together, our results uncovered a unique role of ATG9A in trastuzumab resistance (Figure 6) and highlight the clinical relevance of ATG9A as a target in patients where Her2 targeting drugs are no longer effective. However, as this is the first study to establish a new function of ATG9A involvement in trastuzumab resistance development, further work and *in vivo* studies, including use of patient derived xenograft (PDX) models might help us to better understand its behaviour and to validate whether it is an effective target in mice. Future work is also required in order to elucidate the exact mechanism of action of ATG9A-mediated Cbl's activation and its subsequent ability to promote the degradation of Her2. Additional questions include why this effect is observed only in trastuzumab resistant cells, and more importantly, whether this is the case in patients’ tissues as well.

## MATERIALS AND METHODS

### Cell lines, reagents, antibodies and plasmids

BT474, JIMT1 and MDA-453 cells were maintained in Dulbecco's Modified Eagle's Medium (DMEM) supplemented with 10% fetal calf serum (FCS) and 1% penicillin (P)/streptomycin (S)/glutamine (G). BT474 trastuzumab-resistant (BT474-TR) cells were generated by treating parental BT474 cells with increasing concentrations of trastuzumab for 6 months, as described previously [29]. BT474-TR cells were normally maintained in the presence of trastuzumab (10 ug/ml). All cells were incubated at 37°C in humidified 5% CO_2_. FuGENE^®^ HD transfection reagent was obtained from Roche (Burgess Hill, UK). Plasmids containing human wild type ATG9A and empty vector were obtained from OriGene. siRNAs targeting ATG9A (#1: CTGGATCCACCGGCTTATCAA; #2: CACCCGTGGTCTCAAGTACAA) were from Qiagen. Cells were transfected with either siControl or verified siRNAs against the targets (final concentration 40 nM) and HiPerFect reagent according to the manufacturer's instructions (Qiagen) for 72 hours.

Antibodies used: ATG9A (ab108338, Abcam), Her2 (#2165, Cell Signaling Technology), α-tubulin (ab4074, Abcam), Phospho-Akt (Ser473) (#9271, Cell Signaling Technology), Akt (#9272, Cell Signaling Technology), c-Cbl (#2747, Cell Signaling Technology), Phospho-c-Cbl (Tyr731) (#3554, Cell Signaling Technology), Phospho-c-Cbl (Tyr774) (#3555, Cell Signaling Technology), LAMP1 (ab24170, Abcam).

### SILAC cell culture

To generate SILAC conditions (Dundee Cell Products Ltd, Dundee, UK), normal DMEM medium deficient in arginine (R) and lysine (K) was supplemented with stable isotope-encoded arginine and lysine as previously described [30–32]. L-[^13^C_6_] arginine (R6)/L-[^2^H_4_] lysine (K4) and L-[^13^C_6_, ^15^N_4_] arginine (R10)/L-[^13^C_6_, ^15^N_2_] lysine (K8) were used for ‘medium’ and ‘heavy’ labelling respectively. Parental BT474 and BT474 Trastuzumab resistant (BT474-TR) cells were then grown for 7 cell divisions in either R6K4 ‘medium’ or R10K8 ‘heavy’ medium, respectively, supplemented with 10% dialyzed fetal bovine serum (10 kDa MWCO), 1% (10 mg/ml) streptomycin/(10,000 units/ml) penicillin, 2 mM glutamine and 1 mM sodium pyruvate.

### Protein digestion and peptide fractionation

Equal amounts of protein from labelled samples (1:1) were combined prior to protein digestion. Briefly, samples were reduced in 10 mM DTT and alkylated in 50 mM Iodoacetamide prior to boiling in loading buffer, and then separated by one-dimensional SDS-PAGE (4–12% Bis-Tris Novex mini-gel, Invitrogen) and visualized by colloidal Coomassie staining (Novex, Invitrogen). The entire protein gel lanes were excised and cut into 10 slices each. Every gel slice was subjected to in-gel digestion with trypsin overnight at 37°C. The resulting tryptic peptides were extracted by formic acid (1%) and acetonitrile (CH_3_CN), lyophilized in a speedvac and resuspended in 1% formic acid.

### Mass spectrometry analysis

As described previously [32], trypsin-digested peptides were separated using an Ultimate 3000 RSLC (Thermo Scientific) nanoflow LC system. On average 0.5 μg was loaded with a constant flow of 5 μl/min onto an Acclaim PepMap100 nanoViper C18 trap column (100 μm inner-diameter, 2 cm; Themro Scientific). After trap enrichment, peptides were eluted onto an Acclaim PepMap RSLC nanoViper, C18 column (75 μm, 15 cm; Thermo Scientific) with a linear gradient of 2–40% solvent B (80% acetonitrile with 0.08% formic acid) over 65 min with a constant flow of 300 nl/min. The HPLC system was coupled to a linear ion trap Orbitrap hybrid mass spectrometer (LTQ-Orbitrap Velos, Thermo Scientific) via a nano electrospray ion source (Thermo Scientific). The spray voltage was set to 1.2 kV, and the temperature of the heated capillary was set to 250°C. Full-scan MS survey spectra (*m*/*z* 335–1800) in profile mode were acquired in the Orbitrap with a resolution of 60,000 after accumulation of 1,000,000 ions. The fifteen most intense peptide ions from the preview scan in the Orbitrap were fragmented by collision-induced dissociation (normalized collision energy, 35%; activation Q, 0.250; and activation time, 10 ms) in the LTQ Orbitrap after the accumulation of 10,000 ions. Maximal filling times were 1,000 ms for the full scans and 150 ms for the MS/MS scans. Precursor ion charge state screening was enabled, and all unassigned charge states as well as singly charged species were rejected. The dynamic exclusion list was restricted to a maximum of 500 entries with a maximum retention period of 180 seconds and a relative mass window of 15 ppm. The lock mass option was enabled for survey scans to improve mass accuracy [33]. Data were acquired using the Xcalibur software.

### Proteome quantification

The raw mass spectrometric data files obtained for each experiment were collated into a single quantitated data set using MaxQuant [34] and the Andromeda search engine software [35]. Peptide ratios were calculated for each arginine- and/or lysine-containing peptide as the peak area of labelled arginine/lysine divided by the peak area of non-labelled arginine/lysine for each single-scan mass spectrum. Peptide ratios for all arginine- and lysine-containing peptides sequenced for each protein were averaged. Data were normalised using 1/median ratio value for each identified protein group per labelled sample.

### RNA extraction and RT-qPCR

RNeasy kit (Qiagen) was used to isolate total RNA. Reverse transcription was performed using high capacity cDNA reverse transcription kit (Applied Biosystems). RT-qPCR analysis was performed on a 7900HT Thermocycler (Applied Biosystems) using SYBR^®^ Green master mix and certified primers for *ATG9A, HER2* and *GAPDH* from QuantiTect Primer Assay, Qiagen.

### Cell proliferation assay

Sulphorhodamine B (SRB) assay was performed to determine the growth of breast cancer cell lines in 96-well plates. Briefly, after different treatments, cells were fixed by adding 100 μl/well of ice-cold 40% trichloroacetic acid (TCA) to each culture for 1 h in cold room. Plates were then washed 5× times in running tap water. Cells were stained with 100 μl of 0.4% (W/V) SRB (Sigma S-9012) in 1% acetic acid for 30 mins and plates were washed 5 times in 1% acetic acid and left to air dry overnight. On the day of reading plates, bound dye was solubilized by adding 100 μl of 10 mM tris base (pH 10.5) to all the wells. The absorbance (560–580 nm) was measured on a Tecan microplate reader.

### Western blotting

Protein lysates were extracted using RIPA buffer (Sigma) including fresh protease and phosphatase inhibitors and standard western blotting protocol was performed as described before [31].

### Immunoprecipitation assay

Cells were seeded and transfected with indicated constructs including empty vector (pCMV6) or ATG9A (pCMV6-ATG9A) using FuGENE^®^ HD. Cells were lysed and total protein was quantified. IgG or anti-Her2 antibody was pre-incubated with beads for 2 hours to form IP matrix complex (ImmunoCruz^™^ IP/WB Optima B System, Santa Cruz). 2 mg protein lysate was added into the beads and was incubated on a rotator overnight at 4°C. Beads were then washed with RIPA buffer for three times and were heated in SDS loading buffer. Western blotting was performed to detect protein levels using indicated antibodies.

### Immunofluorescence staining

Cells seeded on glass coverslips and fixed in 4% w/v paraformaldehyde at 37°C for 15 min, permeabilized with 0.1% Triton-X for 10 min and incubated in immuno-fluorescent blocking buffer (10% AB-serum in PBS) for 1 h, followed by incubation with specific primary antibodies (LAMP1 (1:100) and Her2 (1:50)). After washing with PBS, coverslips were incubated for 45 min at 37°C with indicated anti-mouse/rabbit-IgG Alexa Fluor^®^ antibodies (Invitrogen). Nuclei were visualized by DAPI staining. Cells were examined on an Axiovert-200 laser scanning inverted microscope (Zeiss) equipped with a confocal imaging system. Image composites of ∼20 × 0.5 μm z-stacks were obtained using Axiovision LE software (Zeiss) as previously described [36].

### FACS analysis

Cells were transfected with empty vector or full-length ATG9A plasmids for 24 hours and collected, washed 3× times in ice-cold PBS, fixed in 70% ethanol at 4°C, and then stained with 50 μg/ml propidium iodide. Cells were analyzed on a LSR II flow cytometer (BD Biosciences) using the FACSDiva 6.0 software (BD Biosciences).

### Kaplan-Meier (KM) plotter analysis

KM Plotter online survival analysis, which has massive gene expression data and survival information of more than 3,000 patients, was used [16]. Gene expression data and relapse free and overall survival information are publically available from the Gene Expression Omnibus (GEO, http://www.ncbi.nlm.nih.gov/geo/) and The Cancer Genome Atlas (TCGA). The expression levels of ATG9A were selected. The relevance on relapse free survival (RFS) in all types of breast cancer and Her2 amplified was assessed.

### Bioinformatics and statistical analyses

The bioinformatic analyses for identifying top hits from SILAC-quantitative proteomics were performed in R [37] and SWI-Prolog [38], using Real [39] for connecting the two systems. Significance B test was performed to characterize the most significantly regulated proteins between BT474 parental and BT474-TR cells (*p* < 0.05) [34]. For functional networking, GO analysis was performed at the level 2 of the three GO domains: biological process (BP) cellular component (CC) and molecular function (MF) [40]. Furthermore, the hyper-geometric test from GOstats package was used to identify GO terms that are enriched in each cell line [41]. For every overrepresented GO term, a network connecting de-regulated genes in this GO term was drawn showing the networks amongst these genes in the STRING protein-protein interactions database [42]. Only edges with a confidence value greater than 500 were included (range: 0–999).

## SUPPLEMENTARY MATERIALS FIGURES AND TABLES




