# Supplementary material for: ATG9A loss confers resistance to trastuzumab via c-Cbl mediated Her2 degradation
Source: Oncotarget. 2016 Mar 30;7(19):27599–612. doi: 10.18632/oncotarget.8504 (PMC5053674; doi:10.18632/oncotarget.8504)
Supplement: Supplementary file 1 [file oncotarget-07-27599-s001.pdf]

ATG9A loss confers resistance to trastuzumab via c-Cbl mediated Her2 degradation

Supplementary Materials

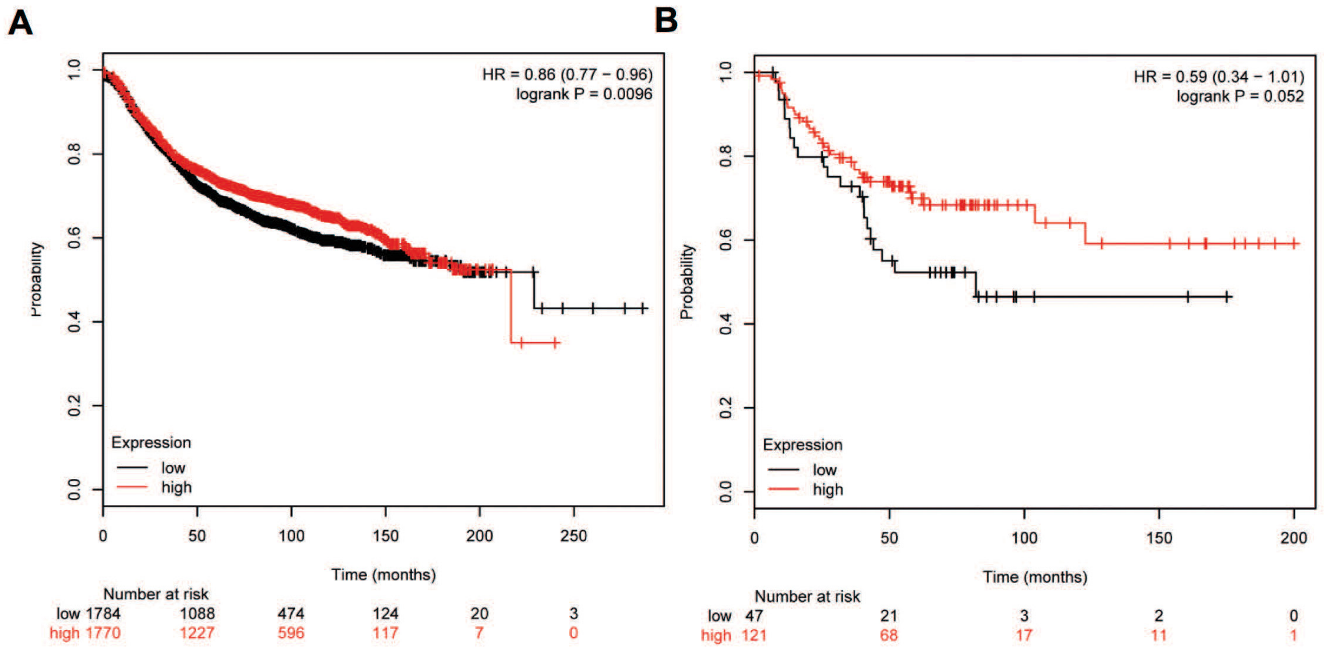

Supplementary Figure S1: Clinical relevance of ATG9A levels in breast cancer patients. The correlations of ATG9A expression levels with relapse free survival (RFS) in breast cancer patients as well as in the subgroup of Her2 amplified breast cancer were assessed using the online survival analysis tool KM Plotter. Our analysis showed that high ATG9A levels were associated with longer RFS in all breast cancer patients (**A**) and interestingly, a similar trend was also observed in patients with Her2 amplification (**B**).

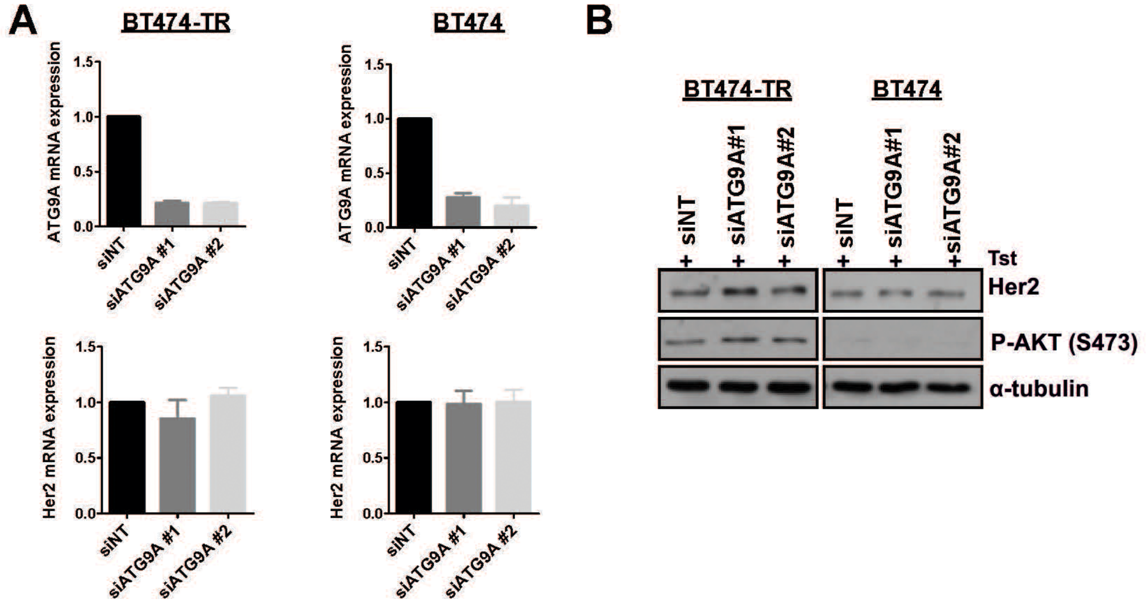

Supplementary Figure S2: Targeting ATG9A increases Her2 protein levels in acquired resistance BT474-TR cells. (**A**) ATG9A and Her2 mRNA (**B**) protein expression in BT474-TR and BT474 cells upon transfection with two different siRNAs against ATG9A or a non-targeting siRNA (siNT) in the presence of trastuzumab (20 µg/ml).

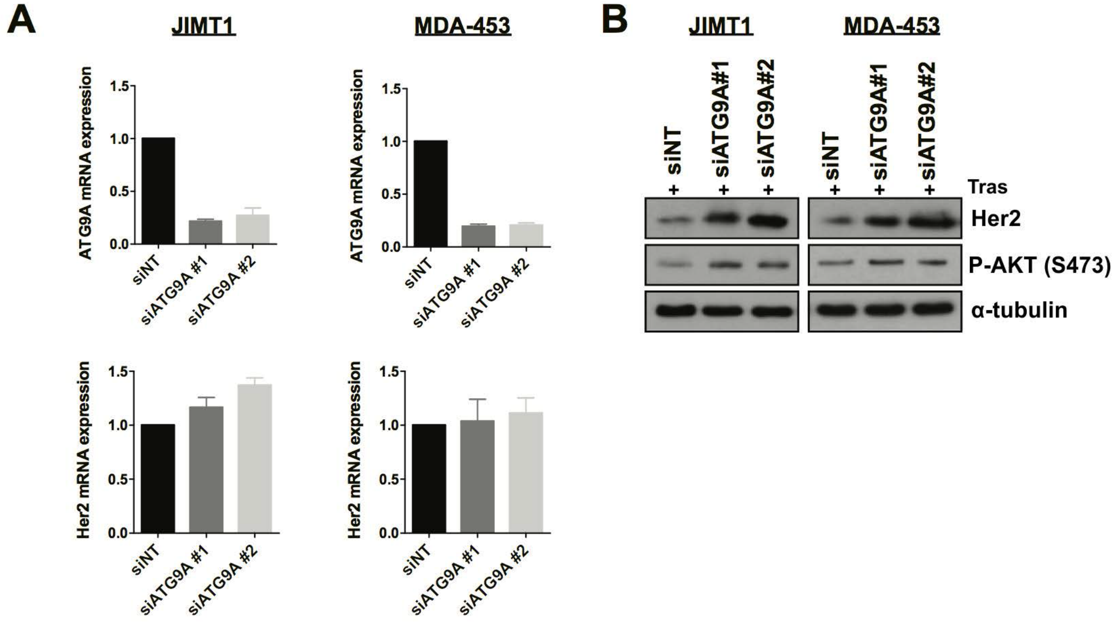

Supplementary Figure S3: Targeting ATG9A increases Her2 protein levels in naturally trastuzumab resistant cells. (**A**) ATG9A and Her2 mRNA (**B**) protein expression in JIMT1 and MDA-453 cells transfected with two different siRNAs against ATG9A or a non-targeting siRNA (siNT) in the presence of trastuzumab (20 µg/ml).

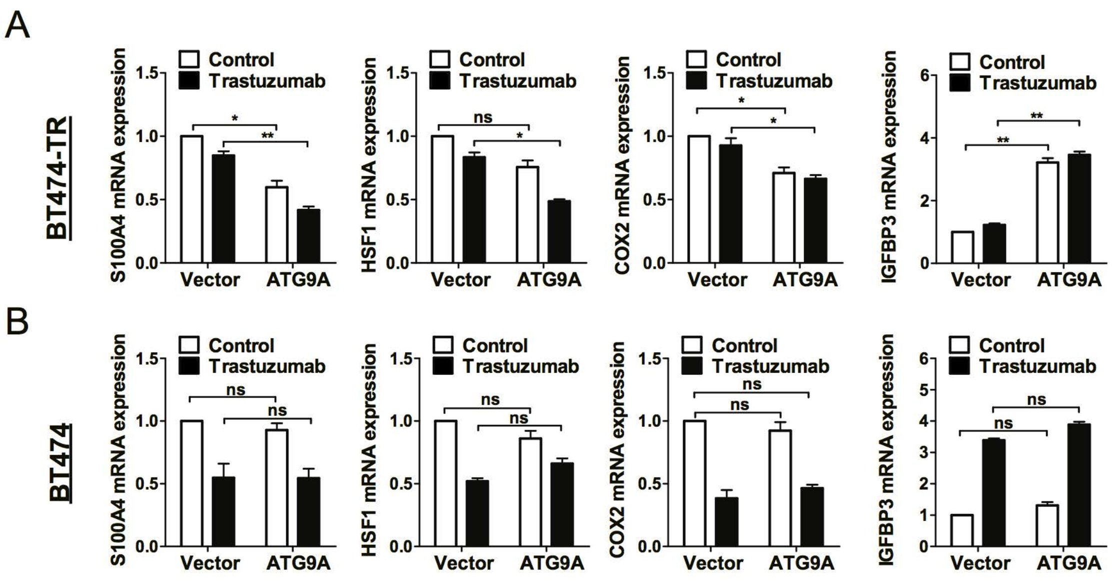

Supplementary Figure S4: ATG9A overexpression affects Her2-regulated genes in acquired resistance BT474-TR cells. (**A**) mRNA expression levels of *S100A4*, *HSF1*, *COX2*, *IGFBP3* in BT474-TR and (**B**) BT474 cells transfected with vector or ATG9A-encoding plasmid in the presence of trastuzumab (20 µg/ml). Data shown are means ± SEM from three experiments. *$P < 0.05$, **$P < 0.01$ and ***$P < 0.001$ unpaired Student's *t* test.

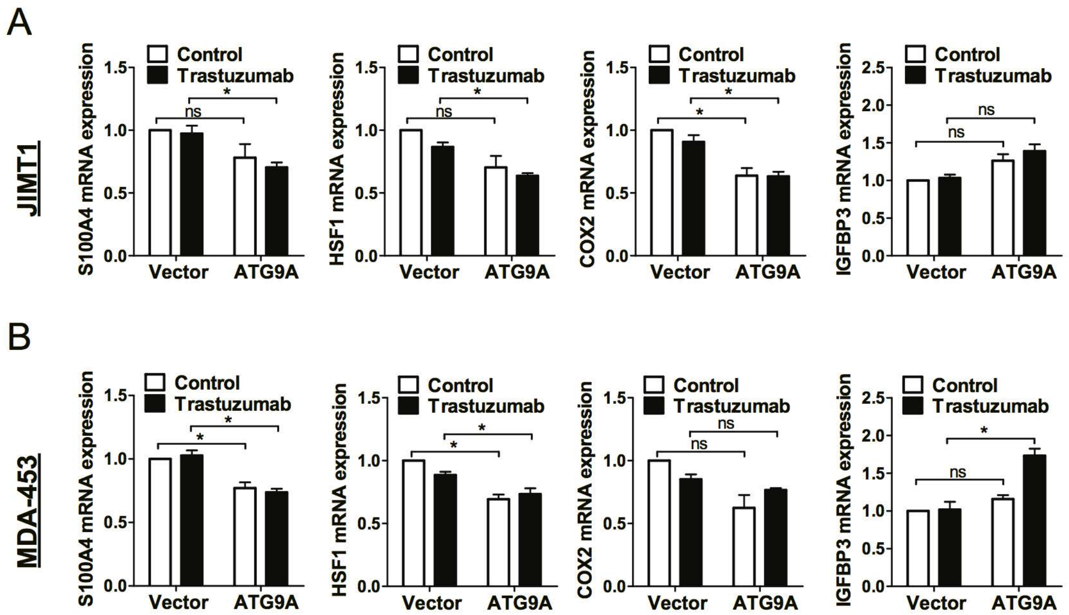

Supplementary Figure S5: ATG9A overexpression affects Her2-regulated genes in naturally trastuzumab resistant cells. (**A**) mRNA expression levels of *S100A4*, *HSF1*, *COX2*, *IGFBP3* in JIMT1 and (**B**) MDA-453 cells transfected with vector or ATG9A-encoding plasmid in the presence of trastuzumab (20 µg/ml). Data shown are means ± SEM from three experiments. *$P < 0.05$, **$P < 0.01$ and ***$P < 0.001$ unpaired Student's *t* test.

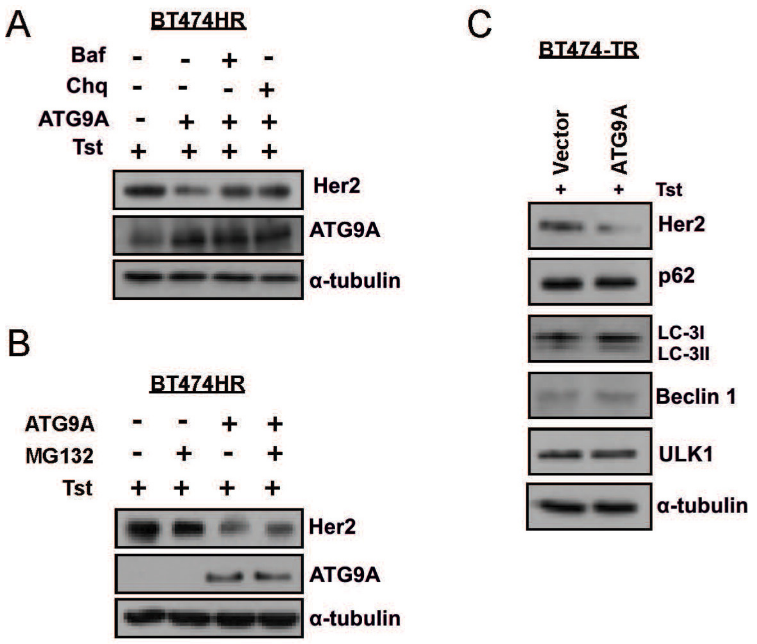

Supplementary Figure S6: Lysosome inhibition abrogates ATG9A-modulated effect on Her2. (**A**) BT474-TR cells were treated with either Bafilomycin (Baf, 0.2 μM, 24 h) or Chloroquine (Chq, 100 μM, 24 h) or (**B**) MG132 (10 μM, 6 h) after transfection with vector or ATG9A-encoding plasmid in the presence of trastuzumab (20 μg/ml). Her2 and ATG9A protein levels were examined by western blotting. (**C**) Autophagy-related proteins p62, LC3, Beclin1 and ULK1 were examined by western blotting in BT474-TR transfected with vector or ATG9A-encoding plasmid in the presence of trastuzumab (20 μg/ml).

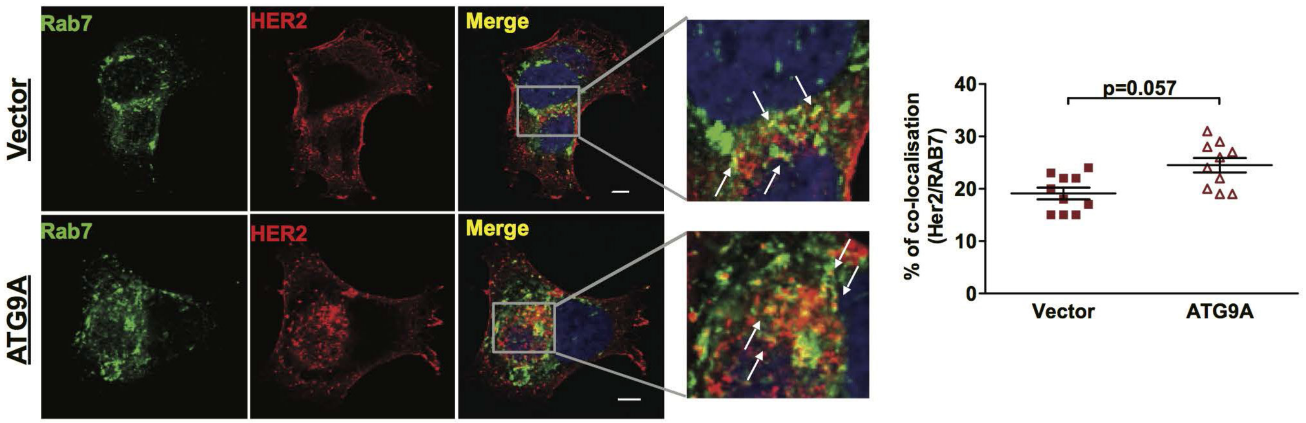

Supplementary Figure S7: ATG9A induces Her2 co-localisation with RAB7 in BT474-TR cells. Immunofluorescence staining of RAB7 and Her2 were analysed in BT474-TR cells transfected with vector-GFP or ATG9A-GFP plasmid in the presence of trastuzumab (20 μg/ml). Percentage of Her2/RAB7 co-localisation was quantified using LEICA LAS AF lite software.

Supplementary Table S1: Protein quantifications of the SILAC analysis
